# The Symptoms and Treatment of Cervical Rib

**Published:** 1913-03-08

**Authors:** 


### The Symptoms and Treatment of Cervical Rib.

The meeting of the Clinical Section, Royal Society of Medicine, on Friday, February 14, under the presidency of Sir William Osler, F.R.S., was largely devoted to the exhibition of a very instructive series of cases of cervical rib, and a subsequent discussion of the whole subject. The chief symptoms manifested by these patients were pain in hand and arm, wasting of muscles, and paralytic symptoms. Some of the patients had had bilateral cervical ribs, and in one of these operation had proved unsatisfactory. In another there were cervical ribs on both sides, but symptoms on one only; and another was improved without operation. Several of the cases shown had pronounced vascular symptoms.

Mr. William Thorburn discussed the surgery of the condition. He said there were three possible great groups of cases in which operation might be called for: (1) those in which it might be indicated for deformity; (2) those in which there were vascular symptoms; (3) those with nervous symptoms. The first class he passed over; and he had not yet been called upon to operate for vascular symptoms alone, though he had removed the rib where vascular and nervous symptoms had co-existed. Those showing nervous symptoms he divided into two great groups, which merged into one another: (1) the neuralgic group, in which the essential symptom was merely pain; (2) the paralytic group, showing paralyses, cramps, atrophies, anasthæsias. The great question was in which groups operation should be done, if done at all. If pain could be relieved by other measures he would deprecate operation for that as a routine. But cases sometimes passed quite rapidly from one group into a more severe one; and on the other hand he had never seen a case which showed a tendency to recovery under any treatment other than operative, though he had seen neglected cases in a crippling stage. In regard to the operation itself, he had tried various incisions, having had to feel his way in the matter in the earlier days; but his decided choice was a long incision straight down, and well back on the neck, over the anterior border of the trapezius muscle. The "collar" incision did not permit of free access to a short, stumpy rib, which might be higher up in the neck than the operator would expect to find it. Still, where the rib was somewhat low, the collar incision might give a better cosmetic result. Using the incision he preferred, he had had but little bleeding; he did a blunt dissection, and struck the rib as near to the vertibral column as possible. He concluded by pointing out the dangers to be avoided, and the necessity for delicate handling of the brachial plexus.

Mr. Percy Sargent also discussed the surgery, narrating cases, and Dr. S. A. K. Wilson and Dr. Farquhar Buzzard dealt with the symptomatology, and showed a number of interesting skiagrams.

